# Supplementary material for: Development of a Highly Specific Monoclonal Antibody-Based Sandwich ELISA for Rapid Detection of Porcine Circovirus Type 3
Source: Viruses. 2025 Oct 5;17(10):1340. doi: 10.3390/v17101340 (PMC12568205; doi:10.3390/v17101340)

Table S1 Primer for Cap protein truncation amplification

Primer	Sequence (5' - 3')	Size (bp)
Cap-N-F	CGCGGATCCATGCGTCATCGTGCGATCTTC	210
Cap-N-R	CCCAAGCTTGGTCAGGCGGGTAATGAAATGATTCG	
Cap-M-F	CGCGGATCCGAATGGGAAACCGCGATTACCTTTG	222
Cap-M-R	CCCAAGCTTACGGCTATGTTTCTTTTTGCTGGTC	
Cap-C-F	CGCGGATCCTATTTCACCCCGAAACCGATCCTG	210
Cap-C-R	CCCAAGCTTGGATTCGTTACAAAAGCGTGCTG	
Cap34-F	CGCGGATCCACCGCGGGTACCTACTACACC	57
Cap52-R	CCCAAGCTTACCAACGGAAATCACGTTCATGGTG	
Cap53-F	CGCGGATCCACCCCGCAGAATAACAAACCGTG	54
Cap70-R	CCCAAGCTTGGTCAGGCGGGTAATGAAATGATTCG	
CapR50	CCCAAGCTTGGAAATCACGTTCATGGTGCTG	150
CapR48	CCCAAGCTTCACGTTCATGGTGCTGTATTTCTTG	144
CapR46	CCCAAGCTTCATGGTGCTGTATTTCTTGGTGTAG	138
CapR44	CCCAAGCTTGCTGTATTTCTTGGTGTAGTAGGTACC	132
CapF36	CGCGGATCCGGTACCTACTACACCAAGAAATACAGC	216
CapF37	CGCGGATCCACCTACTACACCAAGAAATACAGCACC	213
CapF38	CGCGGATCCTACTACACCAAGAAATACAGCACCATG	210
CapF40	CGCGGATCCACCAAGAAATACAGCACCATGAACG	204
CapF42	CGCGGATCCAAATACAGCACCATGAACGTGATTTC	198
Cap107R	CCCAAGCTTGGTATGACCGTACATGGTTTTGGTCTG	
CapR42	CCCAAGCTTTTTCTTGGTGTAGTAGGTACCCGC	126
CapR41	CCCAAGCTTCTTGGTGTAGTAGGTACCCGCG	123

Table S2 Primers for point mutations of antigen epitopes

Primer	Sequence (5’ - 3’)
T37AF	ACAGCTGGCGCATACTACACAAAGAAATACTCCACCAT
T37AR	TAGTATGCGCCAGCTGTGGGCCTCCTAATGAA
Y38AF	TGGCACAGCCTACACAAAGAAATACTCCACCATGAA
Y38AR	TTTGTGTAGGCTGTGCCAGCTGTGGGCCTCCTA
Y39AF	GCACATACGCCACAAAGAAATACTCCACCATGAAC
Y39AR	TCTTTGTGGCGTATGTGCCAGCTGTGGGCCTCC
T40AF	GCACATACTACGCAAAGAAATACTCCACCATGAACG
T40AR	CTTTGCGTAGTATGTGCCAGCTGTGGGCCTCC
K41AF1	CACAGCGAAATACTCCACCATGAACGTCATTTC
K41AR1	GTGGAGTATTTCGCTGTGTAGTATGTGCCAGCTGTGG
K42AF1	CACAAAGGCATACTCCACCATGAACGTCATTTC
K42AR1	GTGGAGTATGCCTTTGTGTAGTATGTGCCAGCTGTG

Table S3 Prediction of B cell linear epitope of PCV3 Cap protein

No.	Peptide	length	score
1	66-VISPAQQTKTM-76	11	0.751
2	124-LAGTTSAHPGQSLFFFSRPT-143	20	0.749
3	7-TAGTYYTKKY-16	10	0.742
4	45-WETAITFEY-53	9	0.737
5	182-RYKSVL-187	6	0.727
6	24-VGTPQNNKP-32	9	0.722
7	163-YVPEKTGMT-171	9	0.634
8	91-NTWLQDD-97	7	0.617
9	110-SKKK-113	4	0.613
10	99-YAESSTRKV-107	9	0.572
11	146-LNTYDP-151	6	0.557

Table S4 Addition test of monoclonal antibody

	OD_{450} value				AI value			
mAb	4G1	9A1	9C1	9A5	4G1	9A1	9C1	9A5
4G1	2.173	2.259	2.246	2.264	0	4.18%	3.62%	4.23%
9A1		2.057	2.074	2.082		0	0.84%	1.16%
9C1			2.016	2.164			0	6.88%
9A5				2.151				0

Figure S1 Detection of antibody titers in mice immune serum

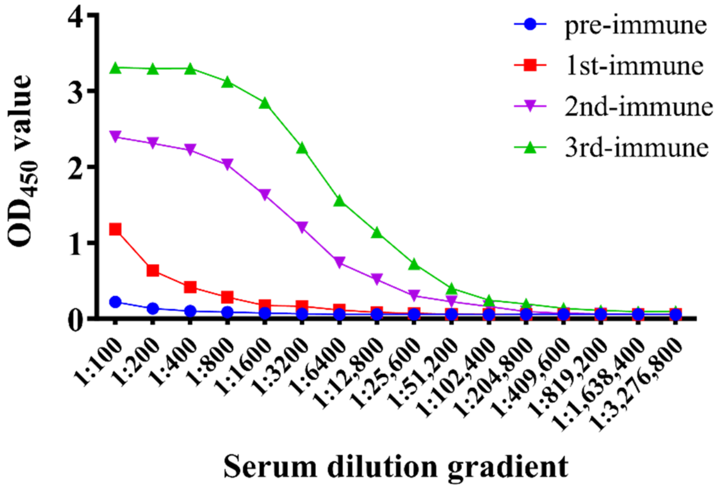

Figure S2 Detection of antibody titers in rabbits immune serum

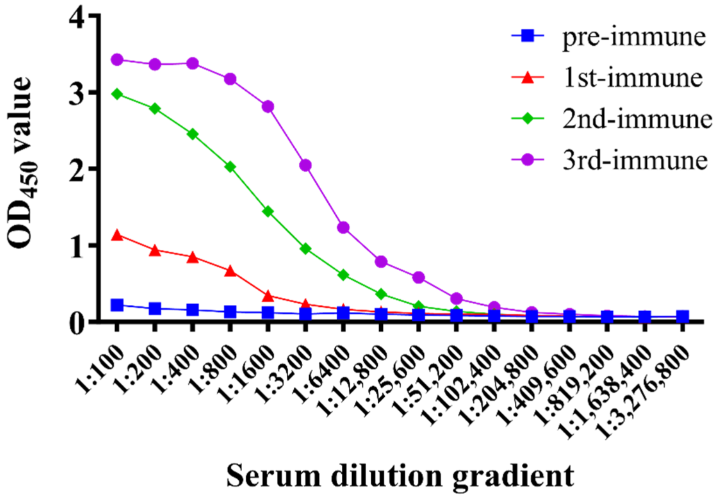

Figure S3 Western blot of the mAb directly incubated with HRP-conjugated goat anti-mouse IgG

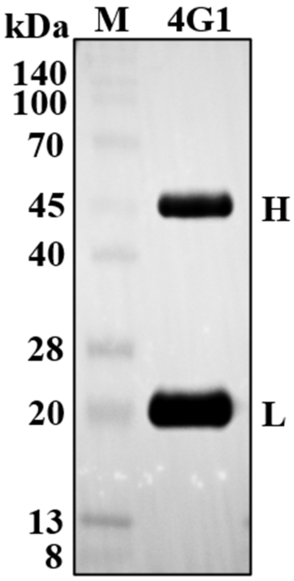

Figure S4 Western blot of the pAb directly incubated with HRP-conjugated goat anti-rabbit IgG

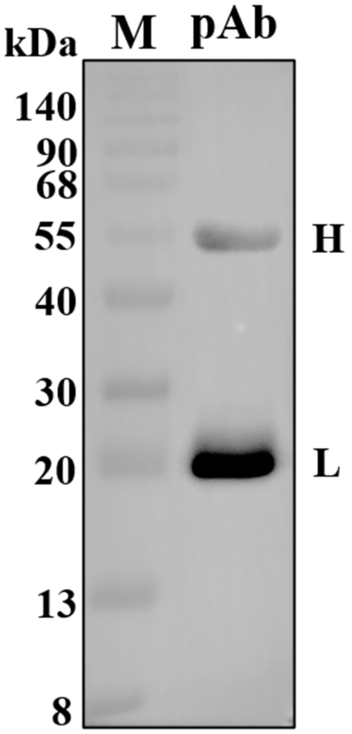

Supplement: Supplementary file 1 [file viruses-17-01340-s001.zip › Supplementary File S1.pdf]
